# Construction and preliminary evaluation of the inpatient glycemic control questionnaire (IGCQ): a survey tool assessing perceptions and knowledge of resident physicians

**DOI:** 10.1186/s12909-019-1657-0

**Published:** 2019-06-24

**Authors:** William B. Horton, Sidney Law, Monika Darji, Mark R. Conaway, Nancy T. Kubiak, Jennifer L. Kirby, S. Calvin Thigpen

**Affiliations:** 1 Divsion of Endocrinology and Metabolism, Department of Medicine, University of Virginia Health System, Charlottesville, VA USA; 2 Department of Medicine, Emory University School of Medicine, Atlanta, GA USA; 3 Division of Endocrinology, Diabetes, and Metabolism, Department of Medicine, University of Chicago, Chicago, IL USA; 4 Division of Translational Research and Applied Statistics, Department of Public Health Sciences, University of Virginia, Charlottesville, VA USA; 5 Division of General Internal Medicine, Palliative Medicine, and Medical Education, Department of Medicine, University of Louisville, Louisville, KY USA; 6 Division of General Internal Medicine and Hypertension, Department of Medicine, University of Mississippi Medical Center, Jackson, MS USA

**Keywords:** Graduate medical education, Hyperglycemia, Physicians, Knowledge, Biostatistics

## Abstract

**Background:**

Uncontrolled hyperglycemia in hospitalized patients, with or without diabetes mellitus, is associated with many adverse outcomes. Resident physicians are the primary managers of inpatient glycemic control (IGC) in many academic and community medical centers; however, no validated survey tools related to their perceptions and knowledge of IGC are currently available. As identification of common barriers to successful IGC amongst resident physicians may help foster better educational interventions (ultimately leading to improvements in IGC and patient care), we sought to construct and preliminarily evaluate such a survey tool.

**Methods:**

We developed the IGC questionnaire (IGCQ) by using previously published but unvalidated survey tools related to physician perspectives on inpatient glycemic control as a framework. We administered the IGCQ to a cohort of resident physicians from the University of Mississippi Medical Center, University of Louisville, Emory University, and the University of Virginia. We then used classical test theory and Rasch Partial Credit Model analyses to preliminarily evaluate and revise the IGCQ. The final survey tool contains 16 total items and three answer-choice categories for most items.

**Results:**

Two hundred forty-six of 438 (56.2%) eligible resident physicians completed the IGCQ during various phases of development.

**Conclusions:**

We constructed and preliminarily evaluated the IGCQ, a survey tool that may be useful for future research into resident physician perceptions and knowledge of IGC. Future studies could seek to externally validate the IGCQ and then utilize the survey tool in pre- and post-intervention assessments.

**Electronic supplementary material:**

The online version of this article (10.1186/s12909-019-1657-0) contains supplementary material, which is available to authorized users.

## Background

Hyperglycemia is common in the inpatient setting and affects up to one-third of patients admitted to general medical and surgical wards [1–3]. Uncontrolled hyperglycemia in hospitalized patients, with or without diabetes mellitus (DM), is associated with adverse outcomes including increased rates of infection and mortality and longer hospital stay [4–7]. Various studies in both critically and noncritically ill hyperglycemic inpatients demonstrate that improved inpatient glycemic control (IGC) can reduce rates of hospital complications, infections, and cost [8–11]. As more than 90% of patients with DM are admitted for reasons unrelated to the disease and may be cared for by staff without specific DM expertise, IGC is often poor [12]. The recent consortium for Planning Research in Inpatient Diabetes (PRIDE) was formed to promote clinical research leading to advancement and improvement of IGC. The consortium outlined eight aspects of IGC which needed to be addressed; the first suggested development and evaluation of provider education tools to improve knowledge of and address barriers to achieving appropriate IGC [13]. Since resident physicians are the primary managers of IGC in many academic and community medical centers, it is important to understand their baseline knowledge and perceptions. Currently, scant data are available [14–17] and no validated survey tools for this topic exist in the medical literature.

Numerous strategies have recently been employed in an attempt to improve IGC, including standardized insulin order sets [18–26], mentoring [27], diabetes care team intervention [28–30], computerized systems [31, 32], physician and nurse education [19, 33, 34], and resident education [35, 36]. While guidelines and interventions designed to improve IGC are gaining attention, knowledge of and barriers to successful implementation of these guidelines along with how they are being translated into clinical practice by resident physicians remains unclear [14]. Identification of common barriers to successful IGC may help foster better educational interventions, ultimately leading to improvements in IGC and patient care. We therefore aimed to construct and evaluate an easy-to-use survey tool for perceptions and knowledge of IGC among resident physicians.

## Methods

### Research locations

We performed this study at four locations: University of Mississippi Medical Center (UMMC), Jackson, MS; University of Virginia Health System (UVA), Charlottesville, Virginia; University of Louisville Health Sciences Center (UL), Louisville, KY; and Emory University Healthcare (Emory), Atlanta, GA.

### Questionnaire design and administration

**Fig. 1:**
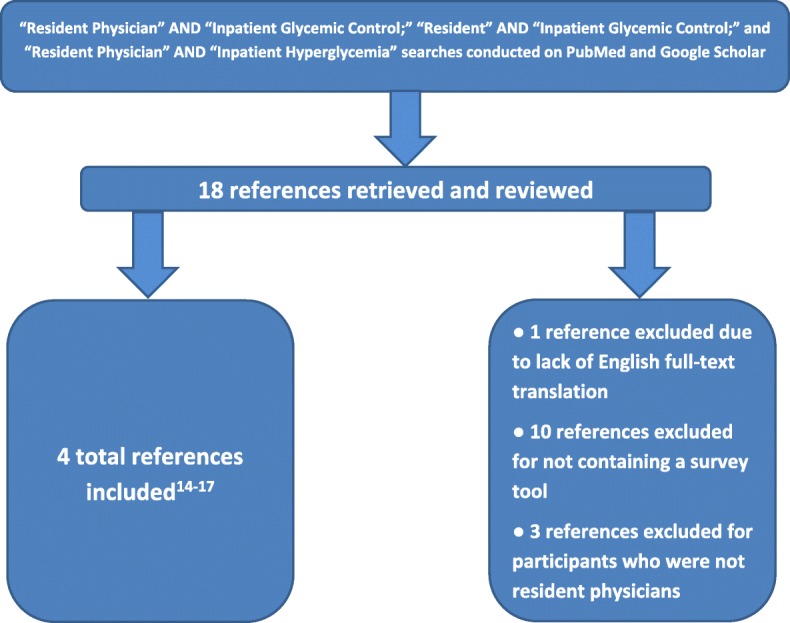
Flowchart for literature review that led to four references being included in the framework for the Inpatient Glycemic Control Questionnaire

To identify relevant prior work on resident physician perspectives of IGC, we searched PUBMED and Google Scholar using search terms “resident physician AND inpatient glycemic control,” “resident AND inpatient glycemic control,” and “resident physician AND inpatient hyperglycemia” (Fig. 1). We then expanded the search by examining citations referenced in the manuscripts initially retrieved. A total of 18 manuscripts were retrieved, four [14–17] of which were deemed highly relevant for inclusion in our study.

The inpatient glycemic control questionnaire (IGCQ) was constructed by using the previously published but unvalidated surveys [14–17] as a framework. We then created a novel survey tool by consolidating and adapting these previously published questionnaires, specifically by decreasing the amount of demographic data collected, expanding the scope and focus of question material, utilizing Likert-scale answer choices, and inserting questions designed to assess knowledge of suggested inpatient glycemic targets from consensus guidelines [4]. Institutional Review Board (IRB) approval at each institution and verbal consent from each participant were obtained  prior to questionnaire administration. The IGCQ was then administered to internal medicine (IM) and medicine-pediatric resident and chief resident physicians to determine their comfort with managing IGC, knowledge of inpatient glycemic target values, and perceived barriers to successful IGC. We distributed questionnaires in person during resident physician educational lectures at UMMC, used Google Forms (Google; San Francisco, California) for data collection at Emory, utilized IRB-approved software (QuestionPro Inc.; San Francisco, California) at UVA, and used SurveyMonkey Pro (SurveyMonkey; San Mateo, California) at UL. Anonymous results were collected during February–May 2015 (UMMC), March–June 2016 (Emory), November–December 2016 (UVA), and March–May 2017 (UL). Survey results were tabulated in an Excel (Microsoft; Redmond, Washington) spreadsheet for data analyses.

### Evaluation methods

#### Rasch partial credit model

RPCM is a unidimensional model that enables “specifically objective” comparisons of persons and items when analyzing responses recorded in two or more ordered categories [37, 38]. RPCM was conducted using Winsteps software (version 3.70.0.5) to examine data for item fitting, dimensionality, and category response functioning (thresholds).

#### Item fitting, dependency, and dimensionality

Fit statistics examine data in comparison with expectations of RPCM. Item fitting is calculated using chi-square statistics and may be reported as mean square (MNSQ), an unstandardized average value of squared differences between the RPCM’s expected and actual values for an item. This value for each item should ideally fall between 0.50 and 1.70 for clinical tools [39]. Item dependencies represent correlation between item difficulties, identifying items potentially measuring the same concept, which could form a sub-dimension and thereby affect overall unidimensionality of the test. Principal component analysis (PCA) of the differences between observed and expected scores or residuals can reveal contrasting items, which can potentially breach the unidimensionality of outcome measure [39].

#### Category response functioning

RPCM compares the probability of a category response to other category responses of the same item as well as category responses from other items [39].

#### Classical test theory

CTT is a traditional quantitative approach to testing the reliability and validity of a scale, based on that scale's individual items. CTT assumes each subject has a *true score*, *T*, that would be obtained if there were no errors in measurement [40]. True scores quantify values on an attribute of interest, defined as the underlying concept, construct, trait, or ability of interest. As values of the true score increase, responses to items representing the same concept should also increase, assuming that item responses are coded so that higher responses reflect more of the concept [40]. We used a Kruskal-Wallis test to evaluate differences in total scores by several variables, including postgraduate year (PGY), program, and gender.

### Preliminary evaluation

#### Pretest (phase 1)

Three IM attending physicians, two endocrinology attending physicians, and two IM resident physicians performed pretest review of the survey tool. Our goals were to ensure that: (1) the IGCQ adequately covered key aspects of both IGC and resident education and (2) question construction was neither too leading nor confusing. Feedback received in this stage raised concerns about two Likert scale questions being “vague and open for interpretation.” Based on these recommendations, we revised the IGCQ accordingly. The IGCQ as presented in Additional file 1: Appendix 1 reflects the survey tool after pretest revision but before distribution to resident physicians.

#### Pilot study (phase 2)

**Fig. 2:**
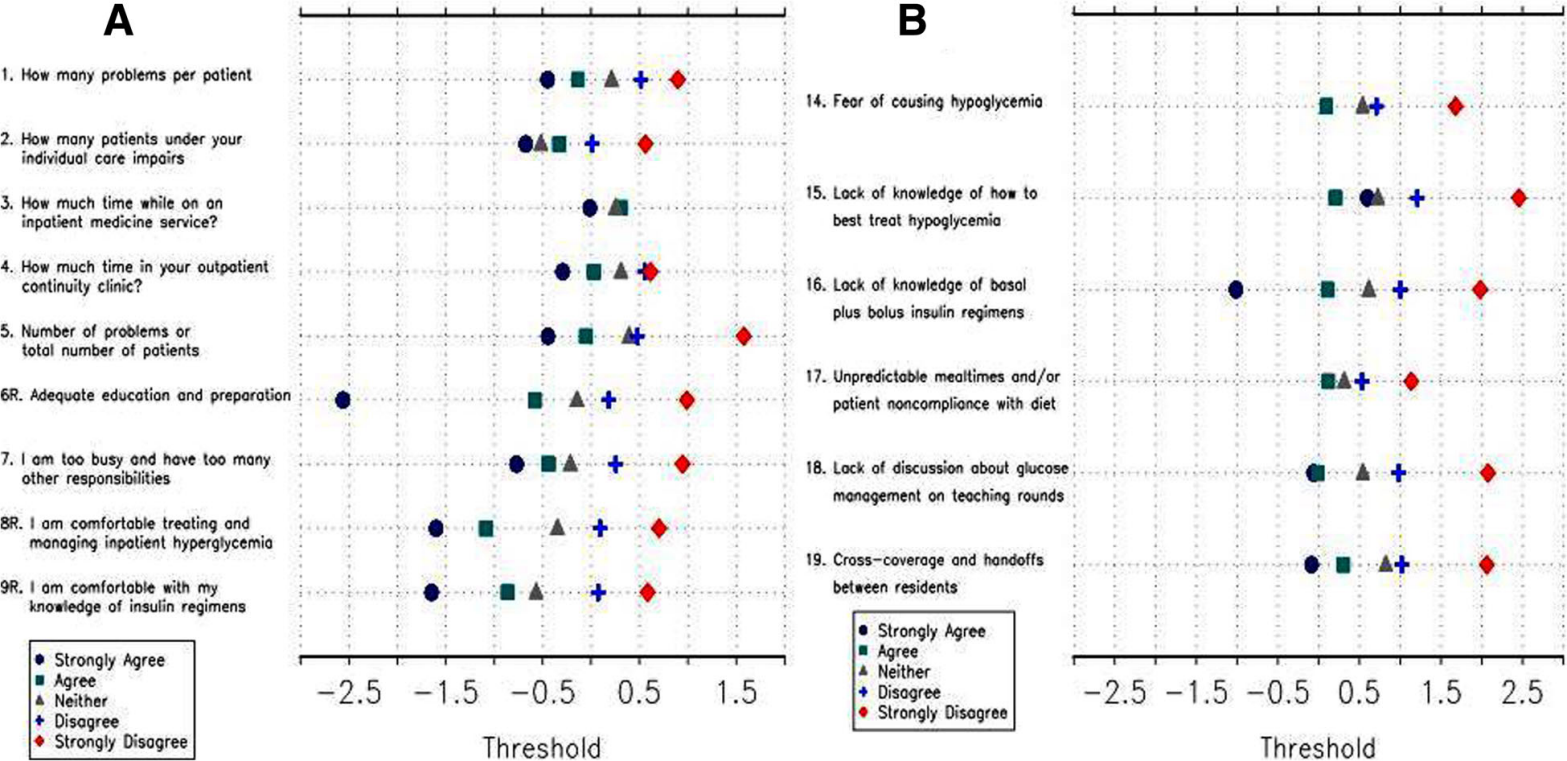
Category response functioning analyses were performed by ordering scales such that if responses to the individual items were summed, higher scores would indicate greater comfort in managing (Questions 1–9) or lower perceived barriers to (Questions 14–19) inpatient glycemic control. Analyses of pilot study (phase 2) data with initial 5-choice answer scale demonstrated notable threshold disorder with little discrimination. Panel **a** demonstrates thresholds for Questions 1–9 and Panel **b** demonstrates thresholds for Questions 14–19

**Fig. 3:**
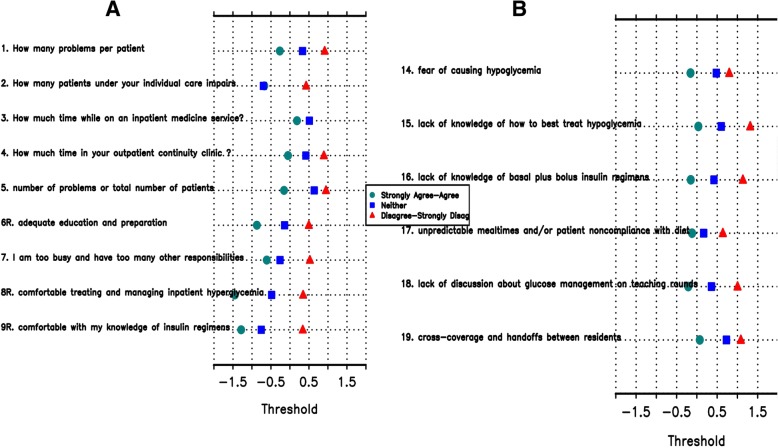
Category response functioning analyses performed on pilot study (phase 2) data with 3-choice answer scale demonstrated much less threshold disorder. Panel **a** demonstrates thresholds for Questions 1–9 and Panel **b** demonstrates thresholds for Questions 14–19

The IGCQ was administered to 182 resident physicians at UMMC, UVA, and Emory. We then used Rasch analysis of collected data to evaluate construct validity of the IGCQ. We did not perform Rasch analysis of knowledge-based items (IGCQ Questions 10–13), as we wanted to preserve five answer choices for these items in order to maintain question complexity and delineate true knowledge of IGC during preliminary evaluation. RPCM analysis demonstrated disordered thresholds for several items using the initial 5-point answer choice scale (Fig. 2). Category responses for non-knowledge based questions were subsequently merged (e.g., 1 instead of 1 and 2, 3 instead of 4 and 5), and RPCM analysis of the merged data showed improved threshold order (Fig. 3) and acceptable fit statistics (Table 1). The improved psychometric performance of merged data led us to modify the IGCQ by reducing the number of category responses for Likert scale questions from five (1 = “strongly agree,” 2 = “agree,” 3= “neither agree nor disagree,” 4 = “disagree,” and 5 = “strongly disagree”) to three (1 = “agree,” 2 = “neither agree nor disagree,” and 3 = “disagree”). We also reduced category responses for Questions 1 and 2 from five (1 = “2–3,” 2 = “4–5,” 3 = “6–7,” 4 = “8–9,” and 5 = “≥10”) to three (1 = “2–5,” 2 = “6–7,” and 3 = “≥8”). Category responses for Questions 3 and 4 were also reduced from five (1 = “< 1,” 2 = “1–2,” 3 = “3–4,” 4 = “5–6,” and 5 = “≥7”) to three (1 = “0–2,” 2 = “3–4,” and 3 = “≥5”).

**Table 1:** Fit statistics for non-medical knowledge questions from pilot study (phase 2) merged data. Item 17 demonstrates mild misfit

Item	MNSQ
1. How many problems per patient do you believe impairs your ability to manage inpatient glycemia?	0.9518
2. How many patients under your individual care do you believe impairs your ability to manage inpatient glycemia?	1.0637
3. How much time (in hours) would you estimate is spent discussing inpatient glycemic control on teaching rounds each week while on an inpatient medicine service?	1.1664
4. How much time (in hours) would you estimate is spent discussing or managing diabetes in your outpatient continuity clinic across one month (4 clinic sessions)?	1.1841
5. As the number of problems per patient or total number of patients under my individual care begins to make me feel uncomfortable, my ability to appropriately manage inpatient glycemia is impaired?	1.0317
6. I feel that I have received adequate education and preparation for managing inpatient glycemia	0.9577
7. I feel that I am too busy and have too many other responsibilities to adequately manage inpatient glycemia as a resident on an inpatient medicine service	1.0455
8. I feel comfortable treating and managing inpatient hyperglycemia	0.9192
9. I feel comfortable with my knowledge of basal plus bolus subcutaneous insulin regimens	1.0437
14. I believe that fear of causing hypoglycemia is a barrier to successful inpatient glycemic control	1.3266
15. I believe that lack of knowledge of how to best treat hypoglycemia is a barrier to successful inpatient glycemic control	0.7871
16. I believe that lack of knowledge of basal plus bolus insulin regimens is a barrier to successful inpatient glycemic control	0.8231
17. I believe that unpredictable mealtimes and/or patient noncompliance with diet is a barrier to successful inpatient glycemic control	1.7944
18. I believe that lack of discussion about glucose management on teaching rounds is a barrier to successful inpatient glycemic control	0.8881
19. I believe that cross-coverage and handoffs between residents is a barrier to successful inpatient glycemic control	1.0438

#### Further study (phase 3)

**Fig. 4:**
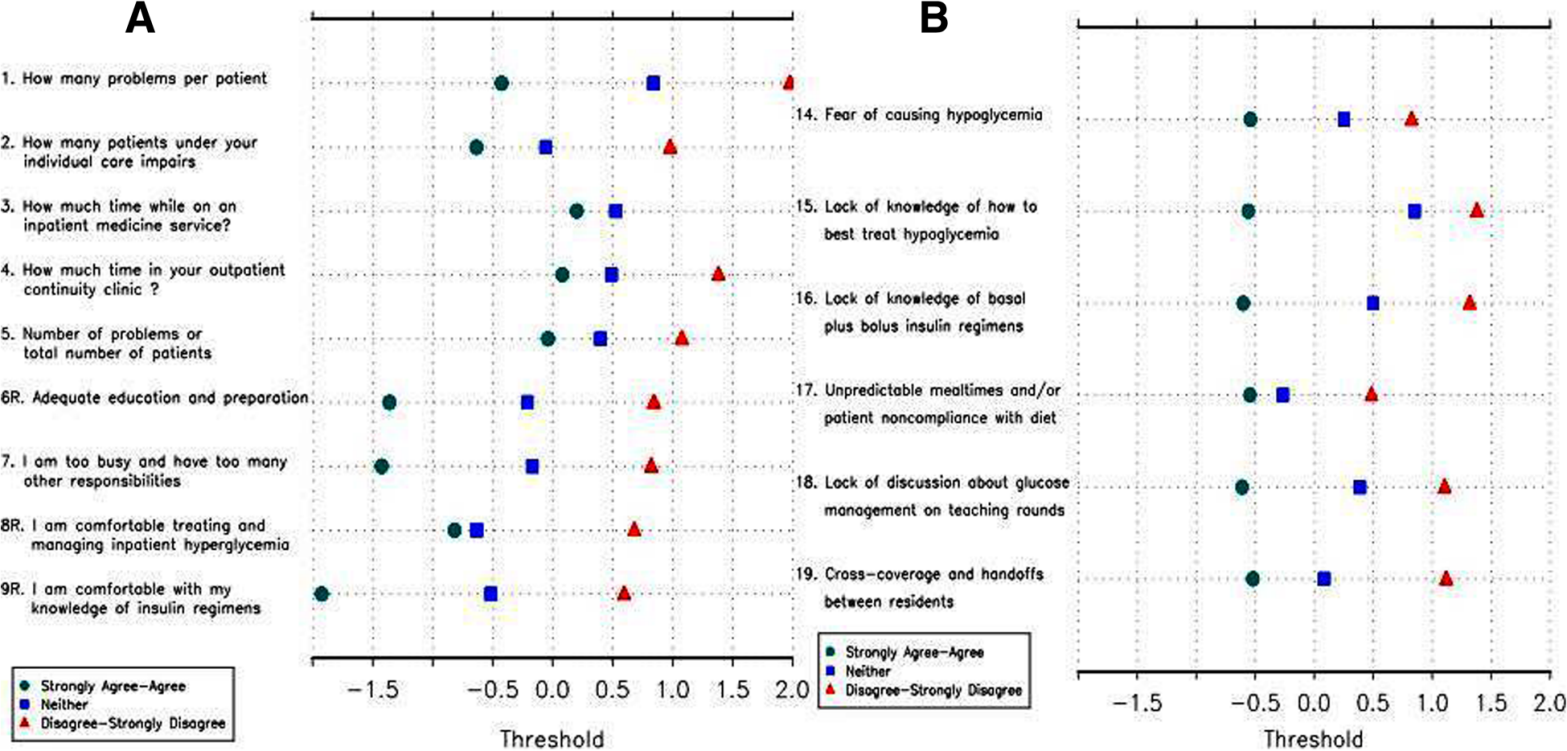
Category response functioning analyses performed on University of Louisville cohort data demonstrated no disordered thresholds. Panel **a** demonstrates thresholds for Questions 1–9 and Panel **b** demonstrates thresholds for Questions 14–19

**Fig. 5:**
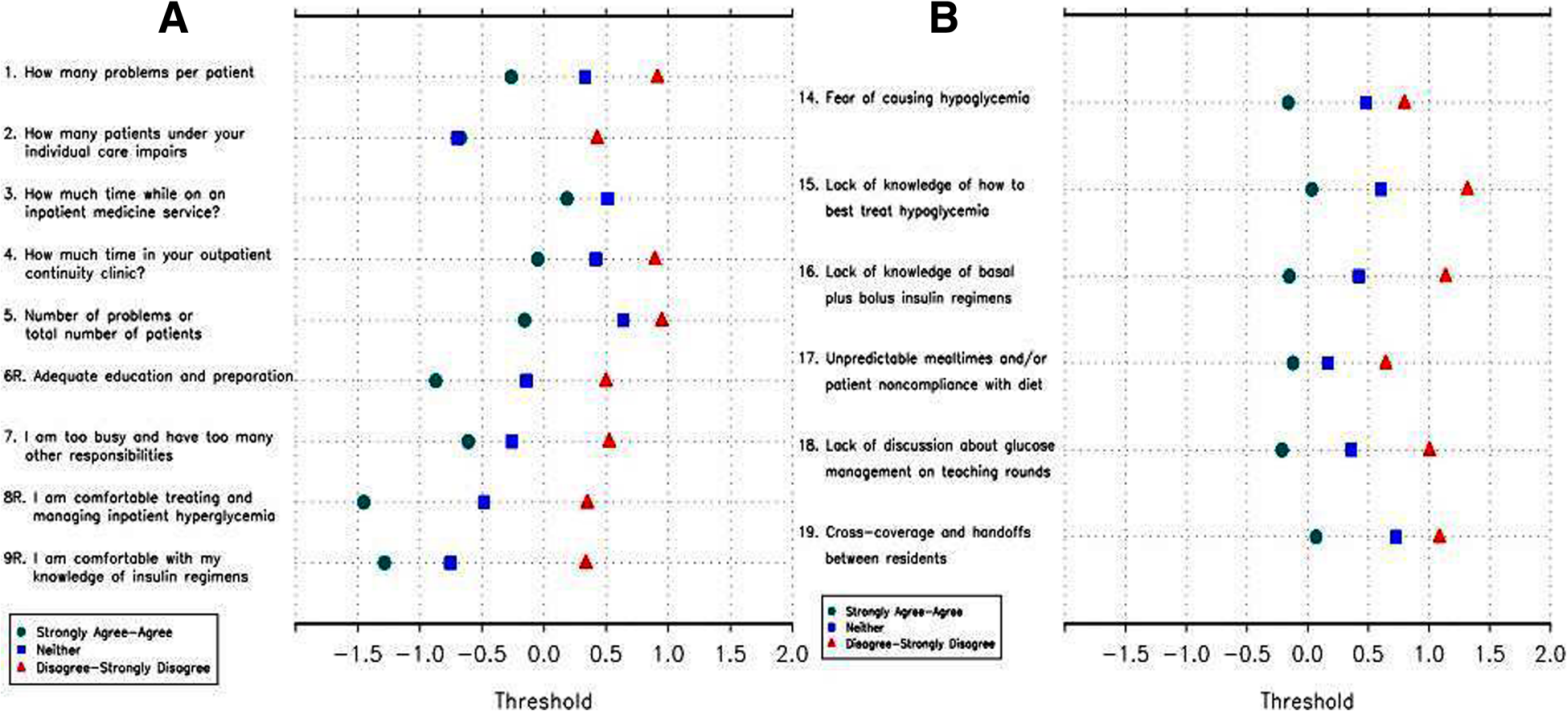
Category response functioning analyses performed on merged data from all four centers demonstrated no disordered thresholds, though items 2 and 3 again showed little discrimination with 3-choice answer scale. Panel **a** demonstrates thresholds for Questions 1–9 and Panel **b** demonstrates thresholds for Questions 14–19

**Fig. 6:**
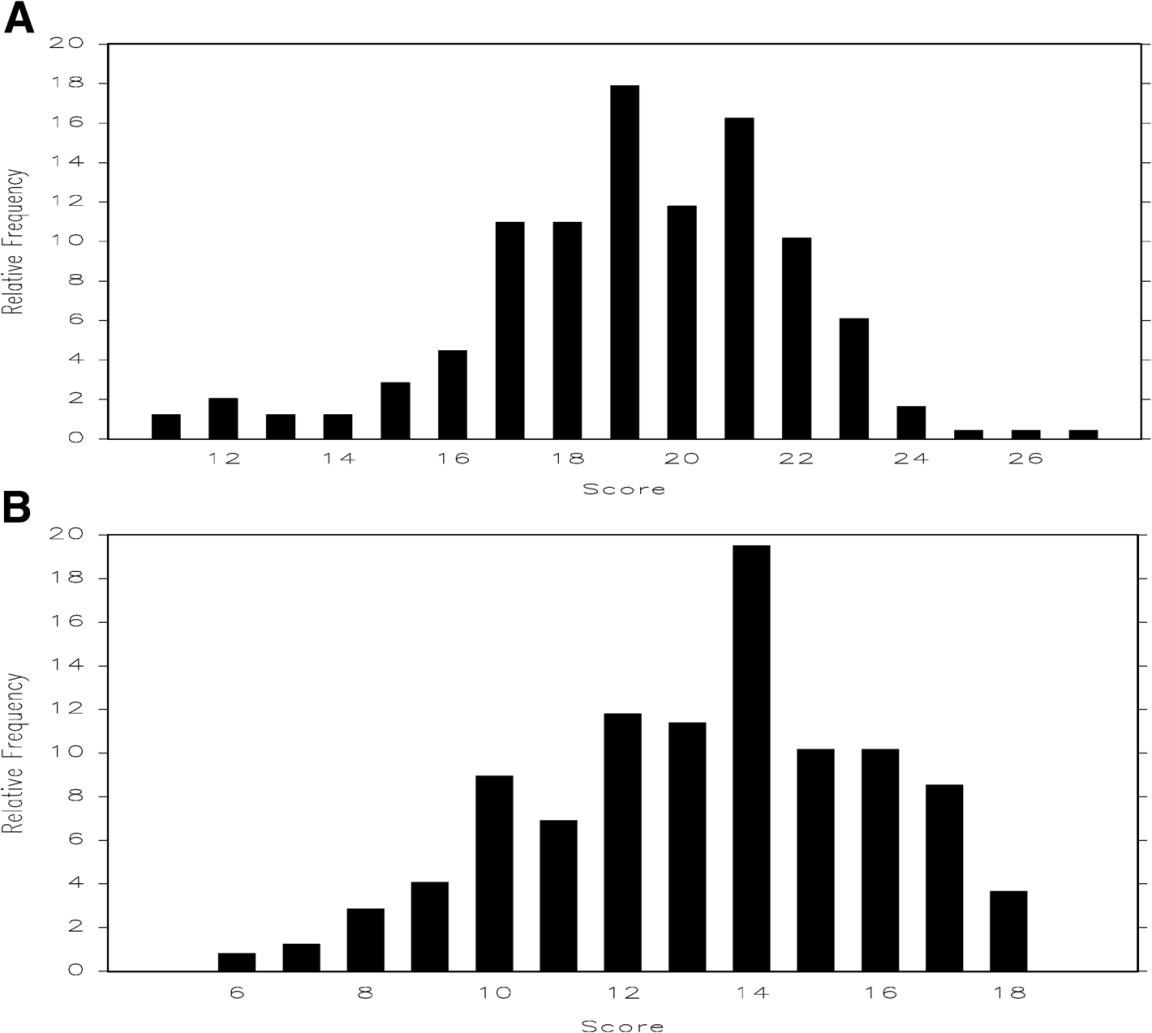
Total score frequencies for the “comfort with managing inpatient glycemic control” (Panel **a**) and “barriers to managing inpatient glycemic control” (Panel **b**) scales

For prospective evaluation of the revised IGCQ, the survey was administered to 64 resident physicians at UL. RPCM was then applied to UL cohort data and revealed no disordered thresholds (Fig. 4), confirming three category responses as the better answer choice scale for non-knowledge based questions. Fit statistics showed improvement in MNSQ for Questions 2 and 3 (Table 2). Questions 4 and 5 trended toward misfit (but not enough to degrade quality of scale) while Question 17 showed severe misfit (Table 2). We then applied RPCM to collective data from all four centers. Analyses demonstrated no disordered thresholds (Fig. 5), though Questions 2 and 3 again showed little discrimination with 3-choice answer scale. Fit statistics demonstrated moderate misfit for Question 17 (Table 3). We also used PCA to assess dimensionality. For Questions 1–9, the first principal component was essentially the average of the items and accounted for 25% of the total variance. A second principal component roughly separated Questions 1–4 from Questions 5–9, indicating that construction of separate scales could be considered. Overall, PCA indicated that it was reasonable to tabulate the responses from Questions 1–9 into a “comfort with managing IGC” scale (with higher scores indicating greater comfort) and Questions 14–19 into a “barriers to IGC” scale (with higher scores indicating lower perception of barriers). For Questions 14–19, the first principal component accounted for 31% of the total variation, which was again essentially the average (without Question 17). The second principal component accounted for 18% of the variation and was mostly due to Question 17, suggesting that the scale would improve if Question 17 were removed. CTT analyses using Kruskal-Wallis test indicated no difference in performance by gender, program, or PGY for the “barriers to IGC” scale. However, the same analyses performed on the “comfort with managing IGC” scale indicated differences in performance by gender and PGY (Table 4). Specifically, comfort with management scores increased as PGY increased (i.e., resident physicians grow more comfortable managing inpatient glycemic control as they progress through training). Figure 6 demonstrates total score frequencies for both scales.

**Table 2:** Fit statistics for non-medical knowledge questions from University of Louisville (phase 3) cohort data. Item 17 demonstrates severe misfit

Item	MNSQ
1. How many problems per patient do you believe impairs your ability to manage inpatient glycemia?	0.4917
2. How many patients under your individual care do you believe impairs your ability to manage inpatient glycemia?	0.9416
3. How much time (in hours) would you estimate is spent discussing inpatient glycemic control on teaching rounds each week while on an inpatient medicine service?	1.1006
4. How much time (in hours) would you estimate is spent discussing or managing diabetes in your outpatient continuity clinic across one month (4 clinic sessions)?	1.8221
5. As the number of problems per patient or total number of patients under my individual care begins to make me feel uncomfortable, my ability to appropriately manage inpatient glycemia is impaired?	1.6467
6. I feel that I have received adequate education and preparation for managing inpatient glycemia	0.7007
7. I feel that I am too busy and have too many other responsibilities to adequately manage inpatient glycemia as a resident on an inpatient medicine service	0.7747
8. I feel comfortable treating and managing inpatient hyperglycemia	1.1287
9. I feel comfortable with my knowledge of basal plus bolus subcutaneous insulin regimens	0.5673
14. I believe that fear of causing hypoglycemia is a barrier to successful inpatient glycemic control	1.3591
15. I believe that lack of knowledge of how to best treat hypoglycemia is a barrier to successful inpatient glycemic control	0.617
16. I believe that lack of knowledge of basal plus bolus insulin regimens is a barrier to successful inpatient glycemic control	0.6946
17. I believe that unpredictable mealtimes and/or patient noncompliance with diet is a barrier to successful inpatient glycemic control	2.568
18. I believe that lack of discussion about glucose management on teaching rounds is a barrier to successful inpatient glycemic control	0.9111
19. I believe that cross-coverage and handoffs between residents is a barrier to successful inpatient glycemic control	0.9631

**Table 3:** Fit statistics for non-medical knowledge questions from multicenter merged data. Item 17 again shows misfit

Item	MNSQ
1. How many problems per patient do you believe impairs your ability to manage inpatient glycemia?	0.8257
2. How many patients under your individual care do you believe impairs your ability to manage inpatient glycemia?	0.9872
3. How much time (in hours) would you estimate is spent discussing inpatient glycemic control on teaching rounds each week while on an inpatient medicine service?	1.1546
4. How much time (in hours) would you estimate is spent discussing or managing diabetes in your outpatient continuity clinic across one month (4 clinic sessions)?	1.5388
5. As the number of problems per patient or total number of patients under my individual care begins to make me feel uncomfortable, my ability to appropriately manage inpatient glycemia is impaired?	1.2367
6. I feel that I have received adequate education and preparation for managing inpatient glycemia	0.8886
7. I feel that I am too busy and have too many other responsibilities to adequately manage inpatient glycemia as a resident on an inpatient medicine service	0.9862
8. I feel comfortable treating and managing inpatient hyperglycemia	0.9315
9. I feel comfortable with my knowledge of basal plus bolus subcutaneous insulin regimens	0.8466
14. I believe that fear of causing hypoglycemia is a barrier to successful inpatient glycemic control	1.3161
15. I believe that lack of knowledge of how to best treat hypoglycemia is a barrier to successful inpatient glycemic control	0.7445
16. I believe that lack of knowledge of basal plus bolus insulin regimens is a barrier to successful inpatient glycemic control	0.7864
17. I believe that unpredictable mealtimes and/or patient noncompliance with diet is a barrier to successful inpatient glycemic control	1.9779
18. I believe that lack of discussion about glucose management on teaching rounds is a barrier to successful inpatient glycemic control	0.8818
19. I believe that cross-coverage and handoffs between residents is a barrier to successful inpatient glycemic control	1.0386

**Table 4:** Differential performance analyses for comfort with managing (IGCQ questions 1–9) and barriers to (IGCQ questions 14–19) inpatient glycemic control scales. *P*-values calculated using Kruskal-Wallis test

Comfort with Managing Inpatient Glycemic Control Scale			
Postgraduate year	Number	Mean Score (SD)	*P*-value
1	84	18.2 (3.1)	
2	83	19.5 (2.5)	
3–4	79	20.0 (2.3)	0.006
Program			
Internal Medicine	223	19.2 (2.7)	
Medicine-Pediatrics	23	19.4 (3.7)	0.87
Gender			
Male	152	19.6 (2.5)	
Female	94	18.5 (3.0)	0.020
Barriers to Inpatient Glycemic Control Scale			
Postgraduate year	Number	Mean Score (SD)	*P*-value
1	84	13.5 (2.9)	
2	83	12.9 (2.5)	
3–4	79	13.4 (2.6)	0.169
Program			
Internal Medicine	223	13.2 (2.7)	
Medicine-Pediatrics	23	13.5 (2.5)	0.67
Gender			
Male	152	13.1 (2.8)	
Female	94	13.6 (2.5)	0.276

*IGCQ* = Inpatient Glycemic Control Questionnaire

#### Final revision (phase 4)

Cumulative RPCM data from all phases of study were considered when making final modifications to the IGCQ. We removed Question 17 as fit statistics demonstrated misfit throughout phases 2–3. We also removed Questions 2 and 3 since RPCM analyses demonstrated threshold disorder in phase 2 testing and little discrimination, even with 3-choice answer scale, in both phases 2 and 3. Finally, Question 4 was removed because its data trended toward mild misfit and because it addressed time spent on education in the outpatient setting, which was ultimately viewed as extraneous in light of the IGCQ’s focus on IGC. These final refinements completed preliminary evaluation and led to the 16-item IGCQ (Additional file 2: Appendix 2). Questions 1–6 represent the “comfort with managing IGC” scale, Questions 7–10 represent the “knowledge of IGC” scale, and Questions 11–16 represent the “barriers to managing IGC” scale.

## Results

### Questionnaire participation

Previous work demonstrated that an estimated minimum sample size range of 108–243 subjects is needed for Rasch analysis for item calibration with ± 0.5 logits at 99% confidence, even if the scale is poorly targeted [41]. However, a minimum sample size of 150 subjects is considered to be adequate in most cases at this confidence level [41]. In our study, 246 of 438 (56.2%) eligible resident physicians completed the IGCQ during various phases of development, including 182 in the pilot study.

## Discussion

To our knowledge, the IGCQ is the first preliminarily evaluated survey tool specifically constructed for assessment of perceptions and knowledge of IGC among resident physicians. Positive attributes of the IGCQ include its basis on previous work in the field, evaluation through RPCM analysis at multiple centers, ease of use, and availability for future research.

The IGCQ is primarily appropriate for use in assessing resident physician perspectives on proper glycemic control of the hospitalized patient. The survey tool has many potential applications and could be used to evaluate the effect of educational interventions on resident physician knowledge of IGC. In the future, it could also be used to compare how resident physician knowledge correlates with real-world clinical care received by hospitalized patients. The IGCQ might also be useful in various other assessments of resident physician perceptions and knowledge. For example, the IGCQ could be easily modified to focus on outpatient glycemic control by reframing Likert scale questions for that specific topic and having knowledge-based questions focus on appropriate outpatient glycemic targets from recent consensus guidelines [42].

Our study has several limitations that should be noted. First, while our results indicate that the IGCQ fits well the Rasch model standards, this analysis is only for one cohort of resident physicians and, thus, further analyses are indicated for future cohorts. Second, each participating institution had different IRB-approved survey software, so we were unable to standardize survey administration across all centers. Third, no differential item functioning (DIF) analyses were performed. DIF occurs in situations where members of different groups show differing probabilities of endorsing an item despite possessing the same level of the ability that the item is intended to measure [43]. Fourth, neither Rasch analysis of knowledge-based questions nor external validation of the survey tool were performed. Future studies could evaluate external validity of the IGCQ and psychometric properties of knowledge-based questions. Further evaluation of any performance differences by gender, ideally with psychometric statistics such as DIF analyses, would also be valuable.

## Conclusion

Herein we presented the construction and preliminary evaluation of the IGCQ. Development of the IGCQ was informed by a review of the current literature on resident physician perspectives of IGC. Examination of the IGCQ utilizing RPCM yielded satisfactory results; however, a few potential issues were identified. We analyzed these issues accordingly and restructured the IGCQ based on study data. The preliminarily -evaluated IGCQ could be valuable for studies seeking to examine the effect of educational interventions on resident physician knowledge of IGC. Future studies could evaluate external validity of the IGCQ and psychometric properties of knowledge-based questions.

## Additional files

Additional file 1: Preliminary version of IGCQ prior to initial evaluation. (DOCX 18 kb)

Additional file 2: Final version of IGCQ after all analyses and revisions were completed. (DOCX 15 kb)

## Data Availability

Please contact the first author for data requests.

## Abbreviations

CTT: Classical Test Theory
DM: Diabetes Mellitus
Emory: Emory University Healthcare
IGC: Inpatient Glycemic Control
IGCQ: Inpatient Glycemic Control Questionnaire
IRB: Institutional Review Board
MNSQ: Mean Square
PCA: Principal Component Analysis
PGY: Postgraduate Year
PRIDE: Planning Research in Inpatient Diabetes
RPCM: Rasch Partial Credit Model
UL: University of Louisville Health Sciences Center
UMMC: University of Mississippi Medical Center
UVA: University of Virginia Health System
