# Supplementary material for: Construction and preliminary evaluation of the inpatient glycemic control questionnaire (IGCQ): a survey tool assessing perceptions and knowledge of resident physicians
Source: BMC Med Educ. 2019 Jun 24;19:228. doi: 10.1186/s12909-019-1657-0 (PMC6591905; doi:10.1186/s12909-019-1657-0)
Supplement: Supplementary file 2 — Final version of IGCQ after all analyses and revisions were completed. (DOCX 15 kb) [file 12909_2019_1657_MOESM2_ESM.docx]

**Inpatient Glycemic Control Questionnaire**

**Demographics**

What is your residency program? ________ Internal Medicine _________ Medicine-Pediatrics

Please indicate PGY: ________1 _________2 ________3 ________4

What is your sex? ________ Male ________ Female

1. How many problems per patient do you believe impairs your ability to manage inpatient glycemia?

**1**. 2-5 **2**. 6-7 **3**. ≥8

1. As the number of problems per patient or total number of patients under my individual care begins to make me feel uncomfortable, my ability to appropriately manage inpatient glycemia is impaired.

**1**. Agree **2**. Neither Agree nor Disagree **3**. Disagree

1. I feel that I have received adequate education and preparation for managing inpatient glycemia.

**1**. Agree **2**. Neither Agree nor Disagree **3**. Disagree

1. I feel that I am too busy and have too many other responsibilities to adequately manage inpatient glycemia as a resident on an inpatient medicine service.

**1**. Agree **2**. Neither Agree nor Disagree **3**. Disagree

1. I feel comfortable treating and managing inpatient hyperglycemia.

**1**. Agree **2**. Neither Agree nor Disagree **3**. Disagree

1. I feel comfortable with my knowledge of basal plus bolus subcutaneous insulin regimens.

**1**. Agree **2**. Neither Agree nor Disagree **3**. Disagree

1. In the hospital, at what glucose level do you first regard your patient as having hypoglycemia?

**1**. <80 **2**. <70 **3**. <60 **4**. <50 **5**. <40

1. In the hospital, what preprandial (pre-meal) glucose level do you target in your non-critically ill patients?

**1**. <200 **2**. <180 **3**. <160 **4**. <140 **5**. <120

1. In the hospital, what random glucose level do you target in your non-critically ill patients?

**1**. <200 **2**. <180 **3**. <160 **4**. <140 **5**. <120

1. In the hospital, what glucose range do you target for your critically ill patients?

**1**. 200-240 **2**. 160-200 **3**. 140-180 **4**. 120-160 **5**. 100-140

1. I believe that fear of causing hypoglycemia is a barrier to successful inpatient glycemic control.

**1**. Agree **2**. Neither Agree nor Disagree **3**. Disagree

1. I believe that lack of knowledge of how to best treat hypoglycemia is a barrier to successful inpatient glycemic control.

**1**. Agree **2**. Neither Agree nor Disagree **3**. Disagree

1. I believe that lack of knowledge of basal plus bolus insulin regimens is a barrier to successful inpatient glycemic control.

**1**. Agree **2**. Neither Agree nor Disagree **3**. Disagree

1. I believe that lack of discussion about glucose management on teaching rounds is a barrier to successful inpatient glycemic control.

**1**. Agree **2**. Neither Agree nor Disagree **3**. Disagree

1. I believe that cross-coverage and handoffs between residents is a barrier to successful inpatient glycemic control.

**1**. Agree **2**. Neither Agree nor Disagree **3**. Disagree

1. Please list the **ONE** factor that you believe is the greatest barrier to successful inpatient glycemic control:

_______________________________________________________________________

**We appreciate you taking time out of your day to complete this survey. Thank you for your participation.**
